# Atrial fibrillation and its arrhythmogenesis associated with insulin resistance

**DOI:** 10.1186/s12933-019-0928-8

**Published:** 2019-09-26

**Authors:** Yi-Hsin Chan, Gwo-Jyh Chang, Ying-Ju Lai, Wei-Jan Chen, Shang-Hung Chang, Li-Man Hung, Chi-Tai Kuo, Yung-Hsin Yeh

**Affiliations:** 1 Cardiovascular Department, Chang-Gung Memorial Hospital, Linkou, Taoyuan Taiwan; 2 College of Medicine, Chang-Gung University, Taoyuan, Taiwan; 3 Microscopy Core Laboratory, Chang-Gung Memorial Hospital, Linkou, Taoyuan, Taiwan; 4 Center for Big Data Analytics and Statistics, Chang-Gung Memorial Hospital, Taoyuan, Taiwan; 5 Graduate Institute of Clinical Medical Sciences, Chang-Gung University, Taoyuan, Taiwan; 6 Department of Biomedical Sciences, College of Medicine, Healthy and Aging Research Center, Chang-Gung University, Taoyuan, Taiwan; 7 Department of Respiratory Therapy, Chang-Gung University College of Medicine, Taoyuan, Taiwan

**Keywords:** Atrial fibrillation, Insulin resistance, CaMKII, TGF-β

## Abstract

**Background:**

Insulin resistance (IR) is considered as a risk factor for atrial fibrillation (AF) even before diabetes develops. The pathophysiology and underlying mechanism are largely unclear.

**Methods:**

We investigated the corresponding mechanism in two IR models of rats fed 15-week high-fat (HFa) and high-fructose/cholesterol (HFr) diets. AF was evaluated and induced by burst atrial pacing. Isolated atrial myocytes were used for whole-cell patch clamp and calcium assessment. Ex vivo whole heart was used for optical mapping. Western blot and immunofluorescence were used for quantitative protein evaluation.

**Results:**

Both HFa and HFr rat atria were vulnerable to AF evaluated by burst atrial pacing. Isolated atrial myocytes from HFa and HFr rats revealed significantly increased sarcoplasmic reticulum calcium content and diastolic calcium sparks. Whole-heart mapping showed prolonged calcium transient duration, conduction velocity reduction, and repetitive ectopic focal discharge in HFa and HFr atria. Protein analysis revealed increased TGF-β1 and collagen expression; increased superoxide production; abnormal upregulation of calcium-homeostasis-related proteins, including oxidized CaMKIIδ, phosphorylated-phospholamban, phosphorylated-RyR-2, and sodium-calcium exchanger; and increased Rac1 activity in both HFa and HFr atria. We observed that inhibition of CaMKII suppressed AF in both HF and HFr diet-fed rats. In vitro palmitate-induced IR neonatal cardiomyocytes and atrial fibroblasts expressed significantly more TGF-β1 than did controls, suggesting paracrine and autocrine effects on both myocytes and fibroblasts.

**Conclusions:**

IR engenders both atrial structural remodeling and abnormal intracellular calcium homeostasis, contributing to increased AF susceptibility. The inhibition of CaMKII may be a potential therapeutic target for AF in insulin resistance.

## Background

Atrial fibrillation (AF) is the most common cardiac arrhythmia in clinical settings, affecting ~ 2% of patients worldwide and carrying a significantly increased risk of ischemic stroke and mortality [1]. Diabetes is one of the major risk factors for AF. A large meta-analysis indicated that patients with diabetes have a nearly 40% greater risk of AF than do patients without diabetes [2]. People with diabetes may have diabetic cardiomyopathy and progress to heart failure independent of coronary artery disease or hypertension, which was attributed to various pathological conditions, including hyperglycemia, obesity, and insulin resistance (IR) [3, 4]. IR is a pathological condition in which cells or tissues fail to respond normally to insulin, and it is characterized by a set of signs comprising obesity, increased blood sugar, dyslipidemia, and elevated blood pressure. IR is the common denominator of metabolic syndrome, prediabetes, and diabetes. People who develop type 2 diabetes usually pass through earlier stages of prediabetes and IR, although these stages are often undiagnosed. Metabolic syndrome and IR have been suggested as risk factors for incident AF, but they have not been supported by all clinical observations [5–9]. Previous studies have demonstrated the pathological mechanisms of overt hyperglycemia-related atrial remodeling and arrhythmogenesis in animal models of diabetes [10, 11]. The characteristics of IR atria and their arrhythmogenesis have not been completely clarified [12, 13]. Food containing high fat, high sugar, and high cholesterol causes the development of IR and significantly increases the susceptibility to diabetes and the risk for cardiovascular disease. Previously, we established two IR rat models by feeding them high-fat (HFa) and high-fructose/high-cholesterol (HFr) diets [14]. In the present study, we used these models to investigate IR-related atrial remodeling and AF vulnerability. We demonstrated significant remodeling in HFa and Hfr rat atria, including changes in the expression of calcium handling–related proteins, increased interstitial fibrosis, and increased oxidative stress and expression of transforming growth factor (TGF)-β1. Furthermore, we showed that oxidized calcium/calmodulin-dependent protein kinase II (CaMKII) may mediate the AF arrhythmogenesis in rats fed HFa and HFr diets.

## Methods

### Animals and diets

Male Sprague–Dawley rats weighing 150–170 g were assigned to three groups fed one of the following three diets for 15 weeks as previously described: [14] (1) a regular standard diet (5.1% fat, 23.5% protein, and 50.3% carbohydrate; Laboratory Autoclavable Rodent Diet 5010; LabDiet, St. Louis, MO, USA) (control; n = 30), (2) an HFr diet (4% cholesterol, 10.1% fat, 17% protein, and 51.6% carbohydrate, TD03468; Harlan Teklad, Indianapolis, IN, USA) with 10% fructose in drinking water (n = 30), or (3) an HFa diet (45% fat, 20% protein and 35% carbohydrate, D12451; Research Diets, New Brunswick, NJ, USA) (n = 30). For sacrifices, all the experimental rats were under deep anesthesia with 3–4% Isoflurane inhalation. Further, euthanasia was taken place by exsanguinations via massive blood-collection from inferior vena cava. The whole heart was taken out immediately for further examination. This study protocol was approved by the Institutional Animal Care and Use Committee of Chang-Gung Memorial Hospital (approval No. 2015032302) and conformed to the Guide for the Care and Use of Laboratory Animals published by the United States National Institutes of Health.

### Whole-heart Langendorff preparation

The heart was harvested immediately and cannulated through the aorta after the rats were killed. Blood was flushed out by injection of a 30-mL cardioplegic solution composed of 134 mM NaCl, 15 mM KCl, 20 mM NaHCO_3_, 0.9 mM NaH_2_PO_4_, 1.8 mM CaCl_2_, 0.5 mM MgSO_4_, and 5.5 mM glucose. Subsequently, the heart was connected to a Langendorff apparatus and perfused with oxygenated Tyrode’s solution heated to 38.0 °C. The composition of Tyrode’s solution was 134 mM NaCl, 4.5 mM KCl, 0.5 mM MgCl_2_, 2 mM NaH_2_PO_4_, 23 mM NaHCO_3_, 1.8 mM CaCl_2_, and 5.5 mM glucose, equilibrated with 95% O_2_ and 5% CO_2_ to maintain a pH of 7.4 [15].

### Optical mapping for whole-heart electrophysiology

The perfusate was maintained at 38.0 °C with a flow rate of approximately 10 mL/min. After 30 min of stabilization, 3.0 mM calcium-sensitive dye (Rhod-2; Life Technologies, Carlsbad, CA, USA) and 10 mM voltage-sensitive dye (RH237; Life Technologies) were added to the perfusate. The heart was washed for 30 min, followed by the addition of 20 mM excitation–contraction uncoupler (blebbistatin; Tocris Bioscience, Minneapolis, MN, USA). A laser light at 532 nm was used to excite the heart, and the fluorescence signal was collected by two fast-speed charge-coupled device cameras. One camera acquired voltage fluorescence through a 710-nm long-pass filter, whereas the other camera simultaneously recorded calcium fluorescence through a 585- to 620-nm bandpass filter. Fluorescence images were captured at a frame rate of 2 ms/frame and 100 × 100 pixels as well as a spatial resolution of 0.35 × 0.35 mm^2^/pixel for 4 s [15].

### Calcium sparks and waves in cardiac myocytes

The isolated atrial cardiomyocytes were harvested and stored in oxygenated Tyrode’s solution. For calcium indicator staining, 10 μL of Fluo-4 AM stock solution was added to 1 mL of cell suspension (final Fluo-4 AM concentration, 10 μM). The cardiomyocytes were placed at room temperature for 20 min to allow for complete de-esterification of AM esters. Ca^2+^ transients were elicited by field stimulation through a pair of platinum electrodes, with a 2-ms suprathreshold square wave voltage pulse delivered by the electrical stimulator at 1 Hz for 30 s to allow a steady-state condition to be reached. A Leica SP8 scanning system (Leica Microsystems, Buffalo Grove, IL, USA) was used for confocal imaging of Ca^2+^ fluorescence. Fluo-4 was excited by the 488-nm line of an argon laser, and emission signals over 505 nm were collected. For recording of Ca^2+^ transients/sparks, a line scan mode was normally used. After sequential scanning, a two-dimensional image of 512 × 1000 lines was generated and stored for offline analysis [16].

### Cellular electrophysiology

Whole-cell patch-clamping was applied for electrophysiological recordings as described previously [17]. The action potential duration (APD) was measured using a patch-clamp amplifier (Axopatch 200B; Molecular Devices Inc., San Jose, CA, USA) and under current-clamp conditions, where APDs were elicited by 3-ms square wave current pulses (intensity, 1 nA) at a rate of 1 Hz and sampled at 10 kHz.

### Western blotting

Proteins were extracted and processed as described elsewhere [18]. Briefly, tissue specimens were lysed in lysis buffer. The proteins were separated using SDS-PAGE and transferred to polyvinylidene fluoride membranes. Subsequently, the membranes were incubated overnight at 4 °C with primary antibodies against transforming growth factor (TGF)-β1, collagen, total Ca^2+^/calmodulin-dependent protein kinase II (t-CaMKIIδ), sodium–calcium exchanger (NCX1), sarco/endoplasmic reticulum Ca^2+^-ATPase II (SERCA II) (Abcam, Cambridge, MA, USA), oxidized CaMKII (ox-CaMKII), phosphorylated phospholamban (p-PLB), total phospholamban (t-PLB) (EMD Millipore, Billerica, MA, USA), phosphorylated ryanodine receptor type 2 (p-RyR2), total RyR2 (t-RyR2), (Proteintech, Rosemont, IL, USA), phosphorylated AKT (p-AKT), total AKT (t-AKT) (Cell Signaling Technology, Danvers, MA, USA), phosphorylated CaMKIIδ (p-CaMKIIδ) (Novus Biologicals, Littleton, CO, USA), and glyceraldehyde 3-phosphate dehydrogenase (GAPDH) (Santa Cruz Biotechnology, Dallas, TX, USA), followed by antirabbit and antimouse immunoglobulin G (Sigma-Aldrich, St. Louis, MO, USA)-conjugated secondary antibodies. Signals were detected through an electrochemiluminescence assay and quantified using densitometry. Immunoreactive signal bands were in the linear range and expressed relative to GAPDH. All the Western blot data were normalized to the expression of GADPH. The phosphrylation sites of proteins were Ser16 on PLB, Ser2814 on RyR and Thr286 on CaMKIIδ. The oxidation site was Met281/282 on CaMKII.

### Immunohistochemistry and detection of intracellular reactive oxygen species

Immunohistochemistry were performed as previously described [19, 20]. Immunohistochemical analyses were performed using confocal microscopy (Leica TCS SP2; Leica Microsystems) with collagen and TGF-β1 (Abcam) as primary antibodies, followed by fluorescein isothiocyanate (green)- or cyanine 3 (red; Chemicon/Fisher Scientific, Pittsburgh, PA, USA)-conjugated secondary antibodies. Nuclei were visualized using DAPI staining. The expression of target proteins was calculated as the protein-occupied area in the tissue divided by the nucleus area. Oxidative stress from reactive oxidative species in the tissues was measured using a fluorescent dye (dihydroethidium [DHE]) and was detected through confocal microscopy. Samples were preincubated with and without 10 μmol/L DHE for 30 min. DHE was excited at 543 nm with an argon laser, and emission at greater than 590 nm was recorded. Two-dimensional images (512 × 512 pixels) were acquired and analyzed using MetaMorph software (Molecular Devices).

### Programmed electrical stimulation

Transvenous atrial stimulation was performed as described previously, with modification [21]. Briefly, the rats were anesthetized with an intraperitoneal injection of ketamine (100 mg/kg) and xylazine (5 mg/kg). All the experimental rats were anesthetized with isoflurane (2–3%) using a small animal ventilator (SAR-830/AP, CWE Inc. USA.) via an inhalation mask. A 1.9-French octapolar electrode catheter (Transonic Systems Inc., Ithaca, NY, USA) was used and attached to the inside wall of the right atrium through the jugular vein. A stimulus amplitude of 2 × diastolic capture threshold with a stimulus duration of 1-ms bursts was used with an automated stimulator (IX-TA; iWorx Systems, Inc., Dover, NH, USA). The pretest burst with a pacing cycle length (PCL) of 100 ms was sustained for 30 s to ensure the capture of atrial stimulation, and pacing bursts with a cycle length of 11.5 ms for 60 s were repeated eight times. AF was defined as a period of rapid irregular atrial rhythm lasting for more than 2 s. If one or more bursts in the two series of bursts provoked an AF episode, AF was considered to be inducible. AF inducibility was expressed as a ratio of pacing-triggered AF episodes/10 pacing bursts in each rat. The AF duration was expressed as the longest period during an AF episode in each rat.

### Rac1 activity

Rac1 activity was determined using a Rac1 activation assay kit (17-283; EMD Millipore). Briefly, tissues were lysed in magnesium lysis buffer (MLB) supplemented with a protease inhibitor mixture. Tissue lysates were precleared for 10 min with glutathione *S*-transferase beads. Lysates were then incubated with p21-activated protein kinase 1 p21-binding domain agarose for 1 h at 4 °C. Beads were washed three times in MLB, and samples were prepared for electrophoresis by adding 1 × SDS loading dye. Samples were boiled for 5 min and resolved by 12% SDS-PAGE. Proteins were transferred to nitrocellulose membrane, and guanosine-triphosphate-bound Rac1 was identified using anti-Rac1 antibody.

### Generation of IR rat atrial fibroblasts and mouse neonatal cardiomyocytes

Fibroblasts were obtained from the atria of adult male Wistar rats (killed with ketamine [100 mg/kg] and xylazine [11.5 mg/kg], administered intraperitoneally) by using collagenase and trypsin digestion and grown in Dulbecco’s modified Eagle’s medium (DMEM) with 10% FBS. Only cells from early passages (1–3) at 80%–90% confluence were used. The medium was replaced with serum-free DMEM for 24 h and subsequently treated with or without glucose (25 mM), insulin (200 nM), and palmitate (50 mM) for an additional 24 h in DMEM with 10% FBS. Neonatal cardiomyocytes were obtained from neonatal mouse heart, and transfer trypsin (50 µg/mL) was placed in a Petri dish and mixed completely, followed by mixture with transfer trypsin inhibitor (2000 µg/mL calcium- and magnesium-free Hanks’ balanced salt solution) in a 37 °C water bath. Collagenase and Leibovitz L-15 media were added and mixed in the tube using a shaking instrument (2–4 rpm) at 37 °C and incubated for 30 to 45 min. The mixture was then triturated 10 times to release cells with a plastic serological pipette. The medium was treated with or without glucose (25 mM), insulin (200 nM), and palmitate (50 mM) for an additional 24 h in L-15 medium with 10% FBS.

### Statistical analysis

All values were expressed as the mean ± SE. Unpaired Student’s *t* test for two groups and one-way ANOVA with post hoc Tukey’s tests for multiple comparisons were applied. Two-way repeated measures ANOVA models followed by post hoc Bonferroni test were applied with factors including APD_80_, coefficient of variation (COV) of APD_80_ and CV versus PCL. A value of P < 0.05 was considered to be significant.

## Results

### Characteristics of rats fed the control, HFa, or HFr diet

Table 1 presents the characteristics of the control, HFa, and HFr diet group rats. The homeostatic model assessment (HOMA) is a method of quantifying IR and β-cell function. Both the HFa and HFr group rats showed higher blood pressure, blood sugar, insulin, triglyceride, and cholesterol, as well as increased insulin resistance (increased HOMA-IR), compared with the control rats, by the end of the 15-week feeding period. In contrast to the HFr rats, the HFa rats weighed more (*P *< 0.001) than did HFr and control rats. The heart-to-body-weight ratio was significantly lower in the HFa rats than it was in the control rats. As shown in Additional file 1: Figure S1, after insulin was intravenously administered, the expression of p-Akt and the p-Akt-to-t-Akt ratio in response to insulin were significantly reduced in the atria from the HFr and HFa rats compared with control rats, indicating that both the HFa and HFr hearts were compatible with the condition of IR.

**Table 1:** Baseline characteristics of control, high-fat (HFa) diet-fed, and high-fructose/cholesterol (HFr) diet-fed rats

	Control (n = 30)	HFa (n = 30)	HFr (n = 30)
0-week body weight (g)	305 ± 13.4	297 ± 8.2	293 ± 9.5
12-week body weight (g)	547 ± 55.8	678 ± 49.4*	555 ± 50.2
HW/BW (mg/g)	2.8 ± 0.5	2.4 ± 0.2*	2.8 ± 0.5
HR (bpm)	355 ± 10.2	380 ± 9.5	378 ± 8.5
MAP (mmHg)	77 ± 14.2	98 ± 9.9*	101 ± 14.3*
Blood glucose (mg/dL)	164 ± 19.9	196 ± 46.8*	214 ± 46.4*
Insulin (μg/L)	1.8 ± 0.9	4.6 ± 1.5*	4.3 ± 2.3*
Triglyceride (mg/dL)	59 ± 17.7	117 ± 38.8 *	118 ± 37.7*
Cholesterol (mg/dL)	75 ± 12.9	96 ± 9.5*	119 ± 25*
HDL (mg/dL)	27.5 ± 4.3	24.7 ± 3.8	32.8 ± 7.1*
HOMA-IR	24.3 ± 10.5	41.9 ± 12.1*	36 ± 12.5*

*HW* heart weight, *BW* body weight, *MAP* mean arterial pressure, *HDL* high-density lipoprotein

HOMA-IR = glucose (mg/dL) × insulin (mIU/L)/405

*P < 0.05 vs. control

Data are presented as mean ± SE

### AF susceptibility in control, HFa, and HFr diet-fed rats

**Fig. 1:**
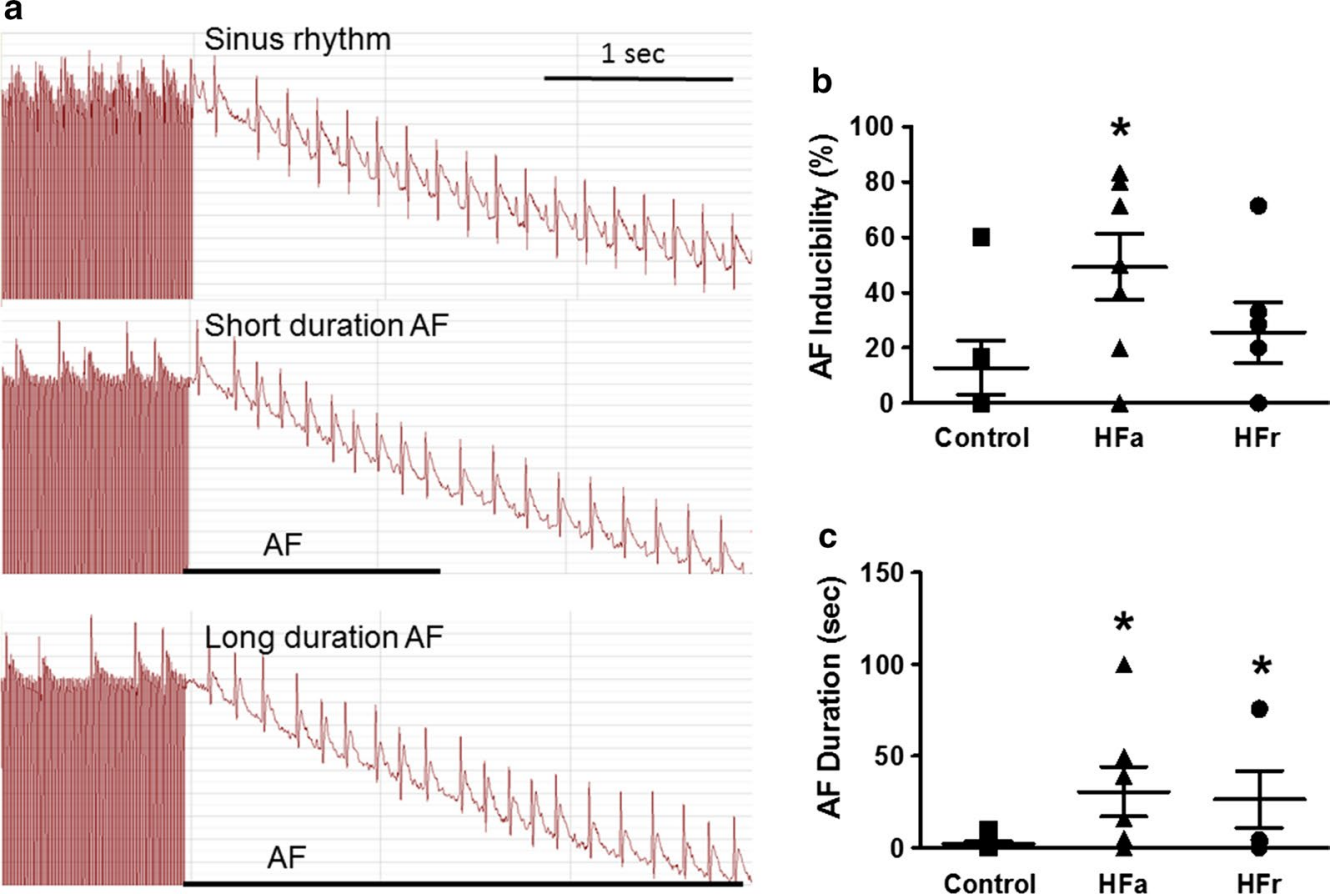
Effect of transvenous atrial burst pacing on atrial fibrillation (AF) inducibility and occurrence in rats fed control, high-fat (HFa), or high-fructose/cholesterol (HFr) diet. **a** Representative examples of electrocardiogram tracing after burst pacing. Upper: sinus rhythm; middle: induced AF with short duration; lower: induced AF with longer duration. **b** Mean ± SE of AF inducibility, which was quantified by the ratio of pacing-induced AF episode in pacing bursts in each rat. The HFa rats had a higher incidence of AF than did the control rats. **c** Mean ± SE of AF duration induced by burst pacing. The duration of AF was quantified by the longest AF among induced episodes in each rat. Both HFa and HFr rats had longer AF duration induced by burst pacing. One-way ANOVA with post hoc Tukey’s tests were applied for comparisons. N = 6 rats for each group. *P < 0.05 versus control

None of the control, HFa, or HFr rats had a spontaneous AF attack at baseline. To evaluate whether the HFa and HFr rats were more vulnerable to atrial tachyarrhythmia, we performed burst atrial pacing using a transvenous approach. As illustrated in Fig. 1, both the HFa and HFr rats were more susceptible to burst-pacing-induced AF than the control rats. Figure 1a presents a representative example of an electrocardiogram of normal sinus rhythm and AF of short and longer duration after burst pacing. The HFa rats exhibited a significantly higher AF inducibility than did the control rats (Fig. 1b). Both the HFa and HFr rats showed a longer AF duration than did the control rats after rapid atrial pacing (Fig. 1c). Taken together, these results suggest that the IR rats were more vulnerable to AF.

### Cellular electrophysiology and intracellular calcium

**Fig. 2:**
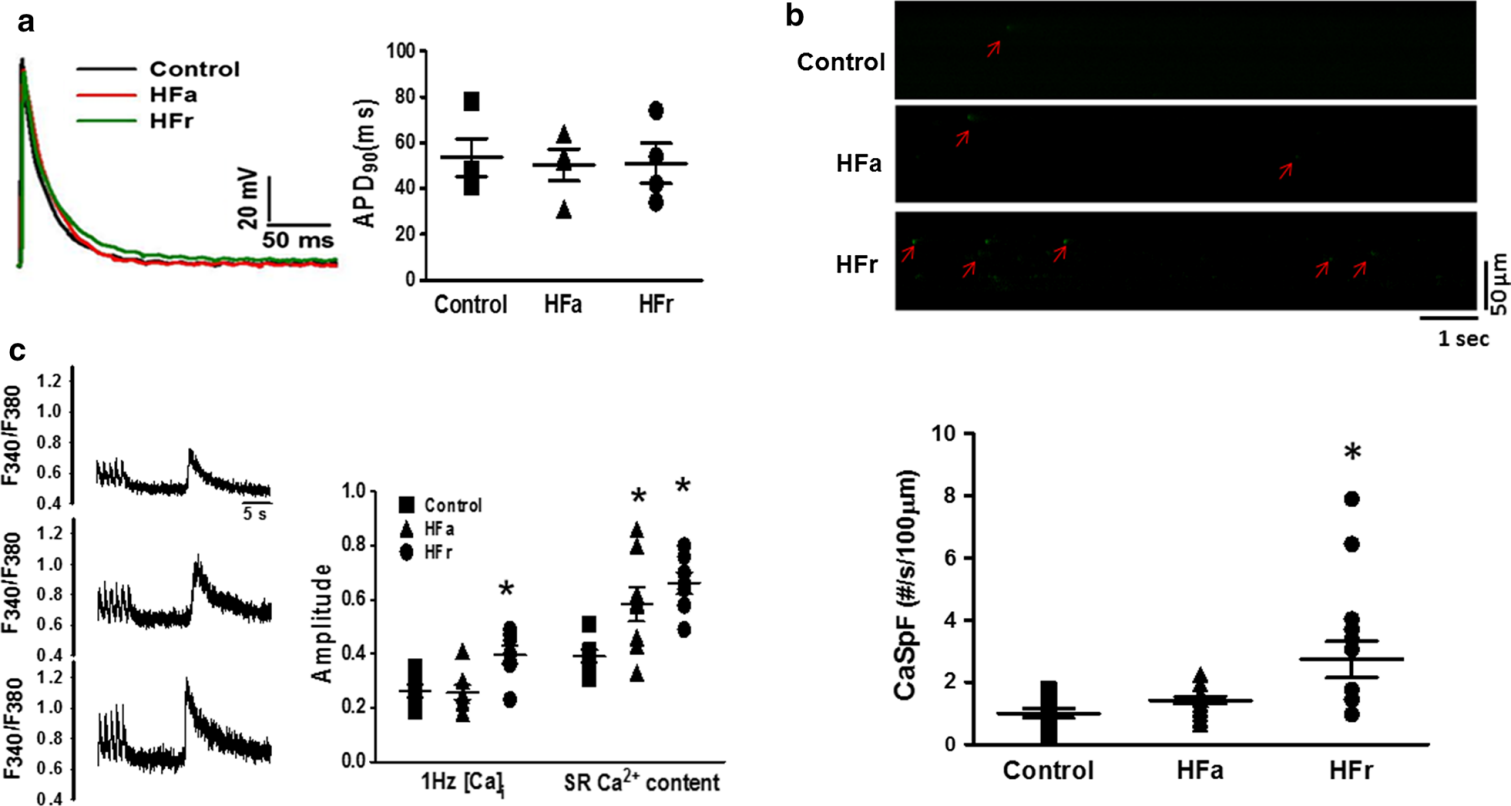
Action potential duration (APD) and calcium imaging of isolated atrial myocytes from high-fat (HFa) and high-fructose/cholesterol (HFr) diet-fed rats. **a** Representative examples and mean ± SE of APD of atrial myocytes. **b** Representative examples and mean ± SE of line-scanning imaging of calcium sparks in atrial cardiomyocytes. **c** Representative examples and mean ± SE of caffeine-induced calcium transients in atrial myocytes from control, HFa, and HFr rats. n = 8–10 atrial myocytes for each group. One-way ANOVA with post hoc Tukey’s tests were applied for comparisons. *P < 0.05 vs. control. Abbreviation: **APD**_**90**_ = action potential duration at 90% repolarization

To evaluate whether changes in ion channels and calcium handling were involved in increased AF vulnerability in the HFa and HFr atria, we performed cellular electrophysiology and intracellular calcium transient (CaT) studies in atrial cardiomyocytes obtained from the HFa and HFr rats. The whole-cell patch-clamp study showed that the control, HFa, and HFr atrial myocytes had similar resting membrane potential and action potential duration values at 20% repolarization (APD_20_), 50% repolarization (APD_50_), and 90% repolarization (APD_90_) (Additional file 2: Table S1 and Additional file 3: Figure S2A). To evaluate sarcoplasmic reticulum (SR) calcium leaks and content, isolated atrial myocytes were paced at 1 Hz for 1 min. Calcium leaks were evaluate by the frequency of calcium sparks after pacing and SR calcium content was evaluated after applying 10 mM caffeine. Confocal laser scanning microscopy revealed that the HFr atrial myocytes had a higher frequency of calcium sparks than did the control atrial myocytes (2.74 ± 1.77/s/100 μm vs. 0.99 ± 0.97/s/100 μm for HFr vs. control rat, *P* < 0.001), suggesting increased diastolic calcium leaks in the HFr atria (Fig. 2b). Confocal calcium imaging showed that the CaTs paced at 1 Hz were increased in the HFr atrial myocytes compared with the control myocytes. Both the HFr and HFa atrial myocytes exhibited larger CaT ratios than those of the control cardiomyocytes after the application (0.66 ± 0.11 vs. 0.39 ± 0.06 F/Fo for HFr vs. control rats, *P* < 0.0001; 0.58 ± 0.18 vs. 0.39 ± 0.06 F/Fo for HFa vs. control rat, *P* = 0.015) (Fig. 2c), suggesting that the calcium content within the SR was significantly greater in the HFa and HFr atrial myocytes than it was in the control myocytes.

### Whole-atria electrophysiology

**Fig. 3:**
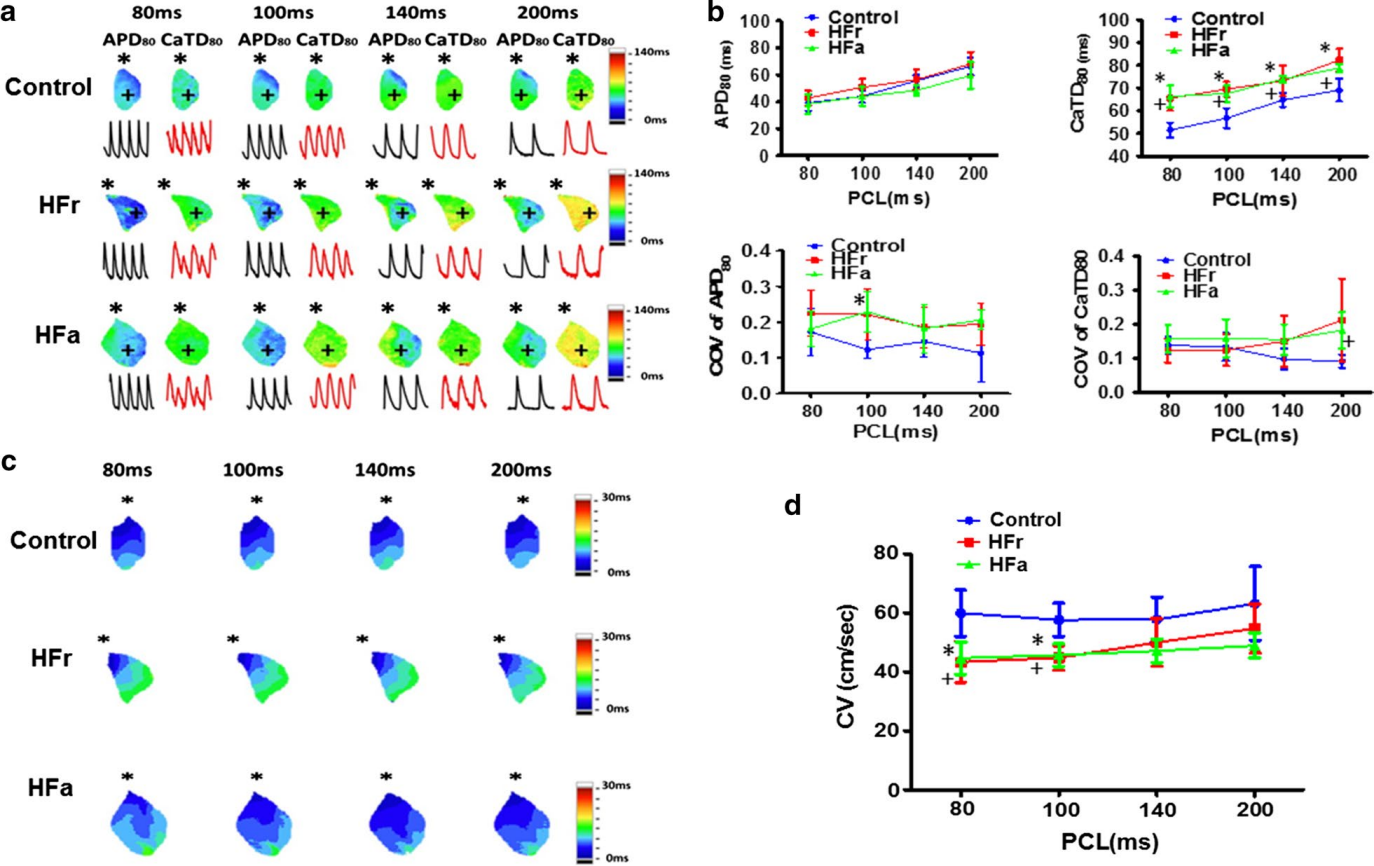
Effects of action potential duration (APD), calcium transient duration (CaTD), and conduction velocity (CV) of right atrial appendages (RAAs) of high-fat (HFa) and high-fructose/cholesterol (HFr) diet-fed rats at different pacing cycle lengths (PCLs). **a** Representative optical membrane voltage (V_m_) and calcium traces, action potential duration at 80% repolarization (APD_80_), and CaTD at 80% repolarization (CaTD_80_) maps of RAAs in control, HFr, and HFa diet-fed rats at different PCLs. Prolongation of overall CaTD_80_ and appearance of calcium alternans of RAAs were noted in HFr and HFa diet-fed rats as compared with control rats. The pacing site (asterisk) was located at the upper edge of the RAA. **b** Mean value of APD_80_ versus PCL for control (n = 5), HFr (n = 5), and HFa diet-fed rats (n = 5). No difference in average APD_80_ was observed between control, HFr, and HFa diet-fed rats. The mean values of CaTD_80_ versus PCL for control, HFr, and HFa diet-fed rats are shown. The RAAs of HFr and HFa diet-fed rats had a larger average CaTD_80_ than did those of the control rats at each PCL. No differences in the mean coefficient of variation (COV) of APD_80_ and CaTD_80_ were observed between control, HFr, and HFa diet-fed rats. **c** Isochronal maps of the representative control, HFr, and HFa diet-fed rats. The pacing site (asterisk) was located at the upper edge of the RAA. **d** Summary of CV measured from the pacing site to the RAA–right atrial junction in control (n = 5), HFr (n = 5), and HFa diet-fed rats (n = 5). The RAAs of HFr and HFa diet-fed rats had a slower CV than did those of control rats at short PCLs of 80 and 100 ms. Two-way repeated measures ANOVA models followed by post hoc Bonferroni test were applied with factors including APD_80_, COV of APD_80_ and CV versus PCL. *P < 0.05 for HFr versus control. ^+^*P* < 0.05 for HFa versus control. Each value represents the mean ± SE of five rats

To evaluate the arrhythmogenic mechanisms of HFa and HFr atria, we performed an optical mapping study. The mapping area was focused on the right atrial appendage (RAA). Figure 3a shows examples of optical APD and CaT duration (CaTD) maps of HFa, HFr, and control RAAs at different PCLs. The HFa and HFr RAAs had comparable action potential duration at 80% repolarization (APD_80_) compared with the control rats at each PCL. At 200-ms PCL, the APD_80_ values for the HFa, HFr, and control RAAs were 59.6 ± 9.8 (n = 5), 68.0 ± 9.1 (n = 5), and 66.4 ± 6.5 ms (n = 5), respectively. At 80-ms PCL, the values were 38.4 ± 7.4, 42.8 ± 5.6, and 39.2 ± 5.9 ms for the HFa, HFr, and control RAAs, respectively. In contrast to the small discrepancy of APD_80_, both the HFa and HFr RAAs showed a significantly longer CaTD at 80% repolarization (CaTD_80_) than did the control rats. At 200-ms PCL, the CaTD_80_ values for the HFa, HFr, and control RAAs were 78.8 ± 1.8, 82.4 ± 4.9, and 69.2 ± 5.0 ms, respectively (*P *= 0.024 for HFa vs. control atria; *P *= 0.002 for HFr vs. control atria). At 80-ms PCL, these values were 66.4 ± 4.8, 65.6 ± 5.5, and 51.6 ± 3.3 ms for the HFa, HFr, and control RAAs, respectively (*P *< 0.001 for HFa vs. control atria; *P *< 0.001 for HFr vs. control atria). In general, the coefficient of variation for either APD_80_ or CaTD_80_ was similar between the HFa, HFr, and control RAAs, suggesting a similar degree of heterogeneity (Fig. 3b). We also observed a lower conduction velocity for the HFa and HFr RAAs than for the control RAA, especially at the shortened PCL of 80 ms (44.7 ± 5.4 vs. 60.0 ± 7.9 cm/s for HFa vs. control atria, *P* = 0.04; 43.4 ± 6.9 vs. 60.0 ± 7.9 cm/s for HFr vs. control atria, *P* = 0.009) and 100 ms (45.7 ± 4.0 vs. 57.7 ± 5.6 cm/s for HFa vs. control atria, *P* = 0.022; 44.8 ± 8.1 vs. 57.7 ± 5.6 cm/s for HFr vs. control atria, *P* = 0.014) (Fig. 3c, d).

### Atrial tachyarrhythmia was mediated by reentry and triggered activities after rapid pacing of HFa and HFr atria

**Fig. 4:**
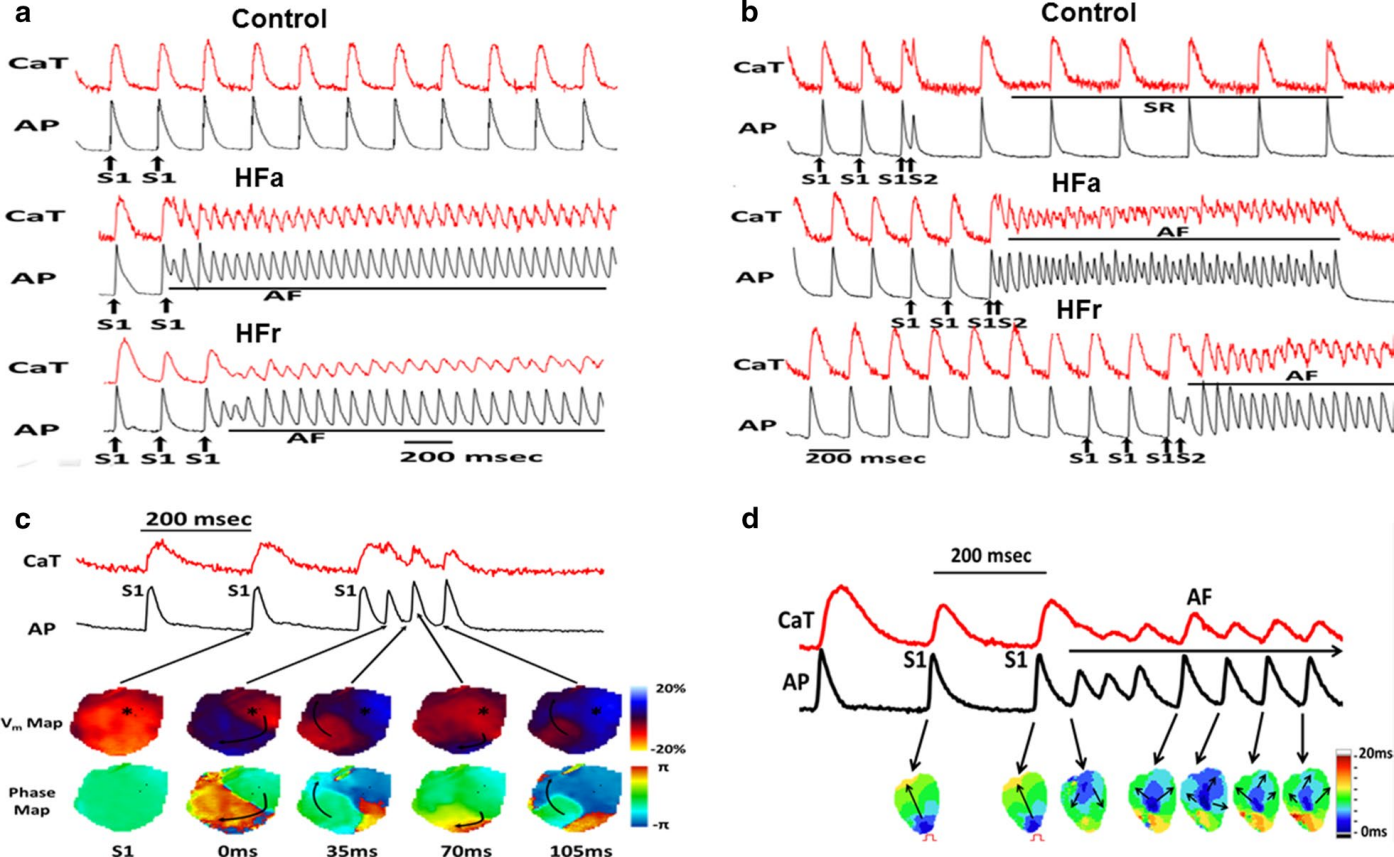
Pacing-induced atrial fibrillation (AF) in high-fat (HFa) and high-fructose/cholesterol (HFr) diet-fed rats. **a** Representative optical membrane action potential (AP) and calcium transient (CaT) recordings in control, HFa, and HFr diet-fed rats under consecutive S1 pacing. **b** Representative AP and CaT traces in control, HFa, and HFr diet-fed rats under consecutive S1 pacing followed by a premature beat of S2. **c** Short run of atrial arrhythmias of one representative HFr diet-fed rat induced by S1 pacing protocol. Notably, the third S1 pacing beat caused the functional conduction block at a distal site, leading to the activation of reentry circle of the right atrial appendage (RAA). **d** Focal discharges of one representative RAA induced by S1 pacing in one HFa diet-fed rat. The isochronal map indicates that the pacing site was in the lower edge of the RAA, whereas repetitive ectopic beats arose from the central region, subsequently spreading to surrounding tissue in the RAA followed by the consecutive S1 pacing

As illustrated in Fig. 4a, in the whole-heart preparation, a spontaneous AF episode was noted in five of nine HFa and four of nine HFr atria during regular pacing at constant PCL (S1). A spontaneous AF episode was noted in only one of nine control atria at the same PCL (*P* = 0.045 for HFa vs. control rats; *P* = 0.114 for HFr vs. control rats). We also attempted AF induction by providing a single premature stimulus (S2) after a train of regular pacing in some atria (S1). The interval between S1 and S2 started at 200 ms and decreased stepwise by 10 ms until AF or an effective refractory period was observed. As presented in Fig. 4b, a single premature stimulation (S2) induced AF in three of three HFa rats and five of five HFr rats; by contrast, it was noted in two of eight control rats (*P* = 0.06 for HFa vs. control rats; *P* = 0.02 for HFr vs. control rats). We observed two mechanisms associated with the initiation and maintenance of AF in HFa and HFr atria. The first mechanism is the reentry. A representative recording of optical mapping in one atrium revealed that S1 pacing captured the tissue near the pacing site. However, the impulse was blocked halfway through the atria, leading to reentry and nonsustained AF episodes (Fig. 4c). Another mechanism is repetitive and ectopic focal discharge. A representative recording of optical mapping in one RAA revealed that consecutive focal-discharge-related sustained atrial arrhythmia arose from the central area of the RAA after the termination of a train of S1 pacing with 200 mL of PCL from the lower edge of the RAA (Fig. 4d). Notably, ectopic focal-discharge-related premature atrial complexes or sustained AF episodes were documented in seven of the nine HFa and seven of the nine HFr atria under either rapid constant S1 pacing or premature stimulus. By contrast, ectopic focal discharge was documented in only one of the nine control atria (*P* = 0.02 for HFa vs. control rats; *P* = 0.02 for HFr vs. control rats).

**Fig. 5:**
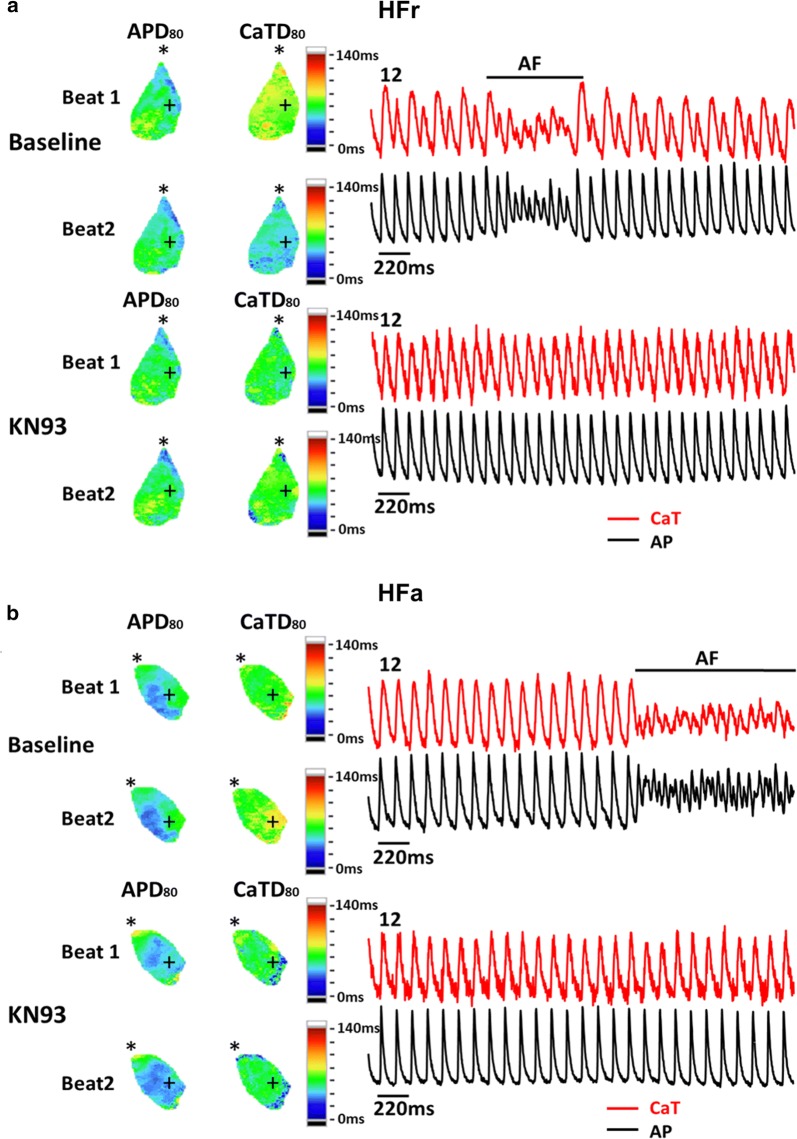
KN-93 suppressed atrial fibrillation (AF) induced by short pacing cycle length (PCL) in high-fat (HFa) and high-fructose/cholesterol (HFr) diet-fed rats. The figure shows representative optical membrane voltage (AP) and calcium transient (CaT), action potential duration at 80% repolarization (APD_80_), and CaT duration at 80% repolarization (CaTD_80_) maps of right atrial appendages (RAAs) in **(*A*)** one HFr diet-fed rat and **(*B*)** one HFa diet-fed rat at short PCLs before and after application of KN-93 (1 μM), respectively. Obvious calcium alternans and short runs of AF were noted in the RAAs of both HFr and HFa diet-fed rats at baseline under rapid pacing. After the application of KN-93 (1 μM), the calcium alternans and AF episode were eliminated under the same PCL. The pacing site (asterisk) was located at the upper edge of the RAA

We also tested whether the inhibition of CaMKII can reduce the inducibility and maintenance of AF in HFa and HFr atria. KN-93 (1 µmol/L), a specific CaMKII inhibitor, was applied in five HFa and five HFr atria. We observed that three of the five HFr and five of the five HFa rats exhibited elimination or increased threshold of AF inducibility after KN-93 infusion (Fig. 5). By contrast, none of the HFr and HFa rats (n = 5 for each group) exhibited elimination of or increases in AF after KN-92 (1 µmol/L) infusion as control (Additional file 3: Figure S2).

### Structural remodeling of the atria of HFa and HFr diet-fed rats

**Fig. 6:**
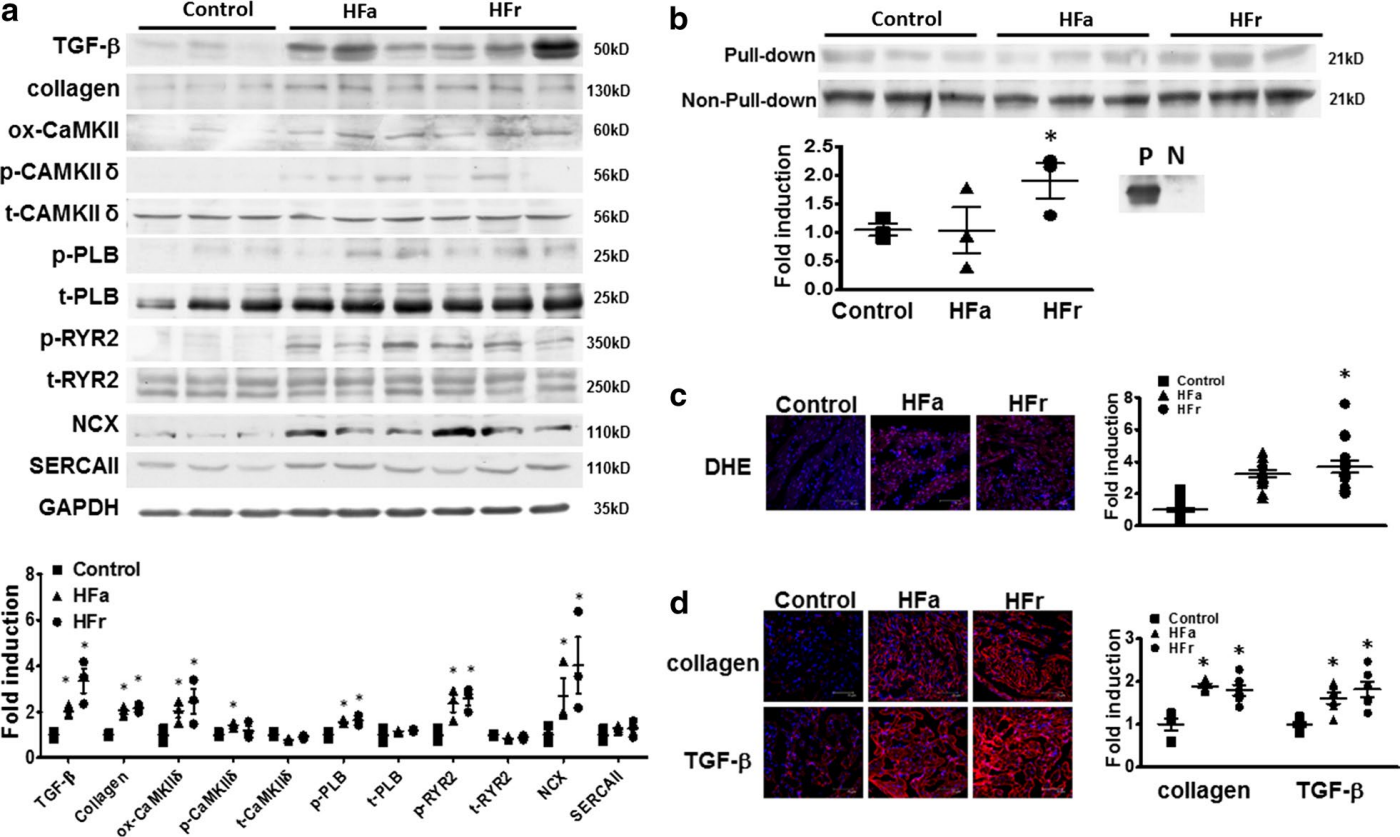
Expression of collagen, transforming growth factor (TGF)-β1, calcium homeostasis-related proteins, Rac1 activities, and superoxide production in high-fat (HFa) and high-fructose/cholesterol (HFr) diet-fed rat atria. **a** Representative examples (upper panel) and mean ± SE of the relative expression levels of each protein quantified by densitometry and normalized to the control level, which was set at 1.0 (lower panel). Each value represents the mean ± SE of four to six independent experiments. **b** Representative examples (upper panel) and mean ± SE of the relative expression levels of Rac1 activities quantified through densitometry and normalized to the control level, which was set at 1.0 (lower panel). Each value represents the mean ± SE of four independent experiments. **c** Evaluation of intracellular superoxide production through the detection of dihydroethidium (DHE). Representative examples (upper panel) and mean ± SE of the relative intensity of immunofluorescence of DHE (lower panel). N = 3 independent experiments. **c** Expression of collagen and TGF-β1 evaluated through confocal immunofluorescence. Representative examples (upper panel). The mean ± SE of relative intensity of immunofluorescence of each protein is normalized to control, which was set at 1.0 (lower panel). N = 3 independent experiments for each group. One-way ANOVA with post hoc Tukey’s tests were applied for comparisons. *P < 0.05 versus control group

TGF-β1 is an important mediator in AF-related structural remodeling [22]. To evaluate whether IR causes structural remodeling and therefore contributes to increased AF vulnerability, we evaluated the expression of TGF-β1 and collagen and the level of superoxide in HFa and HFr atria. Western blot analysis revealed that the HF and HFr atria exhibited increased expression of TGF-β1 and collagen (Fig. 6a). Oxidative stress promotes cardiac fibrosis and arrhythmia-related abnormal calcium handling properties [23, 24]. Confocal immunohistochemistry showed that DHE staining, an indicator of superoxide production, and the expression of TGF-β1 and collagen were significantly increased in the HFa and HFr rats compared with the control rats (Fig. 6c, d). As shown in Fig. 6b, Rac1 activity was significantly increased in the HFr atria, indicating that the increased intracellular oxidative stress likely originated from NADPH oxidase.

### Mechanism of dysregulated calcium handling

To reveal the potential underlying mechanisms of abnormal triggers and increased diastolic calcium leak in IR atria, we evaluated the expression of calcium-homeostasis-related proteins in the HFa and HFr atria. As presented in Fig. 6a, we observed that the expression of calcium-homeostasis-related proteins, including ox-CaMKIIδ, p-PLB, t-PLB, p-RyR2, and NCX1, was significantly upregulated in the HFa and HFr atria, whereas the expression of t-CaMKIIδ, t-RYR2, and SERCAII was similar between the HFa, HFr, and control rat atria. These results suggest that the increased triggered activities observed in the IR atrial myocytes and atria were likely contributed by increased expression of NCX1 and CaMKIIδ-mediated activation of RyR2.

### Expression of TGF-β1 in insulin-resistant cardiac myocytes and fibroblasts

**Fig. 7:**
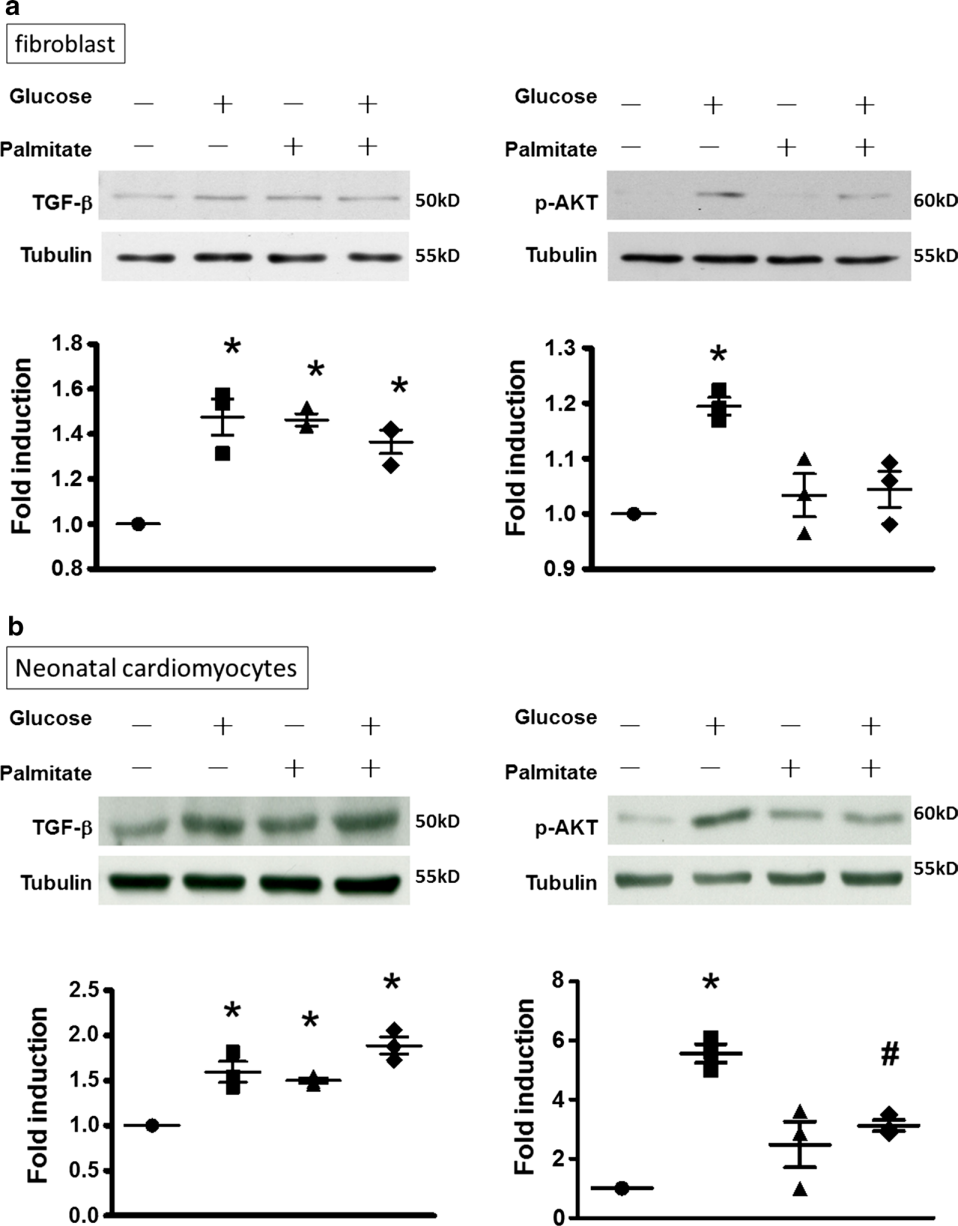
Upregulation of transforming growth factor (TGF)-β1 in insulin-resistant neonatal mouse cardiomyocytes and rat atrial fibroblasts. Representative examples (left panel) and mean ± SE of the relative expression levels of each protein quantified through densitometry and normalized to the control level, which was set at 1.0 (right panel) in **a** atrial fibroblasts and **b** neonatal cardiomyocytes treated with and without palmitate and glucose, respectively. n = 3 independent experiments for each group. One-way ANOVA with post hoc Tukey’s tests were applied for comparisons. *P < 0.05 versus control

To evaluate whether the cardiomyocytes and fibroblasts of IR hearts present factors favoring AF-related pathophysiology, we established in vitro IR models by treating neonatal mouse cardiomyocytes and rat atrial fibroblasts with palmitate, and we evaluated the expression of TGF-β1. As shown in Fig. 7a, b, the expression of p-AKT induced by insulin was significantly attenuated by palmitate in the fibroblasts and cardiomyocytes, respectively, indicating that the insulin signaling pathway was blunted or became resistant to insulin stimulation in the cells. The expression of TGF-β1 was significantly increased in the palmitate-treated atrial fibroblasts and neonatal cardiomyocytes with and without addition of glucose. These results indicate that the insulin-resistant cardiomyocytes and fibroblasts expressed TGF-β1, which may contribute to atrial structural remodeling through paracrine and autocrine pathways.

## Discussion

We provide several lines of evidence demonstrating calcium handling–related and structural remodeling in the rat atria of IR models, which were established by feeding them a 15-week HFa or HFr diet. We showed that the HFa and HFr rat atria were more vulnerable than the controls to AF induced by rapid pacing. We showed abnormal diastolic calcium leak and repetitive ectopic focal discharge in isolated atrial myocytes and whole atria of HFa and HFr rats, suggesting calcium-related triggered activity as the mechanism of AF. The HFa and HFr rat atria presented increased oxidative stress, NADPH oxidase activity, and CaMKII oxidization as well as both calcium handling–related and structural remodeling processes, including increased phosphorylation of RyR and phospholamban and upregulation of collagen and TGF-β1. Upon phosphorylation, the inhibitory effect of PLB on the function of SERCAII is relieved and SR calcium uptake is increased. In our study we showed the SR calcium content was significantly increased both HFa and HFr atria (Fig. 2c), which was likely contributed by hyperphosphorylated PLB. The increased SR calcium content would contribute to calcium leak in isolated atrial myocytes (Fig. 2b) and triggered activities in HFa and HFr atria (Fig. 4). In vitro palmitate-induced IR cardiomyocytes and atrial fibroblasts expressed significantly more TGF-β1 than did the controls, signifying that IR may directly contribute to atrial remodeling independent of hyperglycemia.

### Insulin resistance and atrial remodeling

People with diabetes may have diabetic cardiomyopathy and progress to heart failure independent of coronary artery disease, hypertension, or chronic kidney disease. The pathogenesis of diabetic cardiomyopathy includes impaired myocardial insulin signaling, mitochondrial dysfunction, endoplasmic reticulum stress, impaired calcium homeostasis, abnormal coronary microcirculation, inappropriate neurohumoral activation, and maladaptive immune response [3]. The precise role of IR leading to diabetic cardiomyopathy remains to be clarified. Two major pathways are involved in insulin signaling. The first pathway involves insulin receptor substrate 1 and AKT transduction, which correspond to metabolic response. The second pathway involves mitogen-activated protein kinase (MAPK), which contributes to remodeling changes, including oxidative stress, cardiac hypertrophy, fibrosis, and apoptosis. Our findings imply that the activation of the MAPK pathway likely mediates IR-induced atrial electrical and structural remodeling in HFa and HFr rats.

Recently, Maria et al. reported that in long-term high-fat diet–induced IR rats, insulin-stimulated translocation of glucose transporters 4 and 8 as well as total protein expression was downregulated in the atria [25], which suggests that impaired glucose transport in the atria could provide a metabolic substrate for AF arrhythmogenesis.

We also showed that TGF-β1 was upregulated in the atria of IR rats. The in vitro study suggested that palmitate-induced IR may have a direct effect in TGF-β1 upregulation independent of hyperglycemia or dyslipidemia. TGF-β1 is among the major profibrotic mediators in the atria. The findings are consistent with those of a previous study that pioglitazone, acting as an insulin sensitizer, attenuated atrial fibrosis in a rat model of AF through the suppression of TGF-β1 [26].

### Atrial remodeling in systemic disease and diabetes

In this study, we showed that IR was associated with various aspects of remodeling in the atria, including increased oxidative stress, hyperphosphorylated calcium handling–related proteins, and increased interstitial fibrosis [27, 28]. Remodeling has been demonstrated mostly in the atria from other systemic and cardiovascular disease models, including heart failure, obesity, and coronary artery disease [29]. In general, it would not be surprising that these systemic diseases share common and various AF-precipitating electrical and structural remodeling processes in the atria, with emphasis on increased oxidative stress generated by NADPH oxidase, dysfunctional nitric oxide synthase, and mitochondria [23].

An animal study demonstrated that the diabetic rat atrium induced by streptozotocin had increased interstitial fibrosis, reduced connexin 40 expression, and decreased CV, resulting in APD prolongation and dispersion and increased incidence of APD alternans [10, 11]. Another study revealed that in streptozotocin-induced diabetic rats, adrenergic activation and heterogeneous sympathetic innervation were evident in the diabetic heart, suggesting that neural remodeling may play a crucial role in increased AF vulnerability in diabetes [30].

In contrast to the presentation of the streptozotocin-induced diabetic rats with overt hyperglycemia and severe body weight loss, which favorably mimic the effects of uncontrolled hyperglycemia and type 1 diabetes rather than IR, the HFa and HFr rats presented with ~ 30% elevated blood sugar and 0%–20% increased body weight after 15-week HFa and HFr feeding, which were set for the models of IR and prediabetes. Our findings more favorably reflect the changes in the atria of patients with prediabetes and metabolic syndrome.

### Oxidized CaMKII and AF

We observed increased NADPH oxidase activity in IR rat heart, contributing to increased oxidative stress and upregulated ox-CaMKII. Oxidative stress can induce arrhythmogenic hyperphosphorylation of calcium-handling proteins through the activation of CaMKII. CaMKII hyperactivity has been demonstrated to be causatively associated with AF and ventricular arrhythmias in heart failure through modulating calcium handling–related protein activities [31, 32]. Recently, research revealed that CaMKII increases small-conductance Ca^2+^-activated K^+^ current in atria from patients with AF [33]. A recent study showed ox-CaMKII–mediated ventricular arrhythmia in a mouse model of Duchenne muscular dystrophy [34]. Exposure to hyperglycemia was reported to activate CaMKII in cell culture and a streptozotocin-induced diabetic rat heart [35]. In the present study, we first observed through optical mapping that inhibition of CaMKII activation was effective in preventing AF in the HFa and HFr heart. Inhibition of reactive oxygen species or ox-CaMKII is likely protective against proarrhythmic intracellular calcium handling in IR atria [36].

### Clinical implications

Some antidiabetic medications, including metformin, thiazolidinediones, dipeptidyl peptidase 4 inhibitors, and sodium/glucose cotransporter 2 inhibitor, have been shown to improve myocardial glucose uptake and contractile dysfunction in humans [37] and in an animal model of diabetes [3]. We have shown that both metformin and dipeptidyl peptidase 4 inhibitor decrease the risk of AF in patients with diabetes in a nationwide cohort study [38, 39]. Research revealed that metformin can prevent rapid pacing–induced atrial myocyte structural remodeling by reducing intracellular oxidative stress. Through improvement of IR, these antidiabetic medications may be effective in preventing atrial remodeling and AF in people with IR and prediabetes.

### Study limitations

We could not separately evaluate the effect of IR without conditions of elevated blood pressure, blood sugar, lipids, and cholesterol in these rats, all of which also contribute to atrial remodeling. Rats and humans may have different expression levels of calcium-handling proteins, which may lead to different mechanisms of regulation in calcium homeostasis.

## Conclusions

We conclude that IR promotes factors leading to interstitial fibrosis and abnormal calcium homeostasis in atria, which alter the CV and increase ectopic activities in the atria, contributing to AF genesis. IR may have a direct effect on the expression of TGF-β1 in myocytes and fibroblasts, contributing to atrial fibrosis. These results suggest that upstream therapy targeting CaMKII and TGF-β1 as well as reducing oxidative stress is a potentially effective strategy for preventing AF caused by a diet high in fat, sugar, and cholesterol.

## Supplementary information

**Additional file 1: Figure S1.** Expression of phosphorylated and total AKT in insulin-treated high-fat (HFa) and high-fructose/cholesterol (HFr) diet-fed rat atria. Representative examples (upper panel) and mean ± SE of the relative expression levels of each protein quantified by densitometry and normalized to the control level, which was set at 1.0 (lower panel). Each value represents the mean ± SE of three independent experiments.

**Additional file 2: Table S1.** Cellular electrophysiology in HFa and HFr rats.

**Additional file 3: Figure S2.** Effects of KN-92 in atrial fibrillation (AF) induced by short pacing cycle length (PCL) in high-fat (HFa) and high-fructose/cholesterol (HFr) diet-fed rats. The figure shows representative optical membrane voltage (AP) and calcium transient (CaT), action potential duration at 80% repolarization (APD_80_), and CaT duration at 80% repolarization (CaTD_80_) maps of right atrial appendages (RAAs) in **(*A*)** one HFr diet-fed rat and **(*B*)** one HFa diet-fed rat at short PCLs before and after application of KN-92 (1 µmol/L), respectively. None of the HFr or HFa rat atria (n = 5 for each group) exhibited decrease or increase in AF duration after KN-92 infusion.

## Data Availability

The datasets used and/or analysed during the current study are available from the corresponding author on reasonable request.

## Abbreviations

AF: atrial fibrillation
APD: action potential duration
CaMKIIδ: Ca^2+^/calmodulin-dependent protein kinase IIδ
CaT: intracellular calcium transient
CaTD: duration of intracellular calcium transient
COV: coefficient of variation
DHE: dihydroethidium
DMEM: Dulbecco’s modified Eagle’s medium
NCX: sodium–calcium exchanger
HFa: high fat
HFr: high-fructose/cholesterol
HOMA: homeostatic model assessment
IR: insulin resistance
PCL: pacing cycle length
PLB: phospholamban
RAA: right atrial appendage
SR: sarcoplasmic reticulum
SERCA II: sarco/endoplasmic reticulum Ca^2+^-ATPase II
