# Supplementary figures and images for: Atrial fibrillation and its arrhythmogenesis associated with insulin resistance
Source: Cardiovasc Diabetol. 2019 Sep 26;18:125. doi: 10.1186/s12933-019-0928-8 (PMC6761716; doi:10.1186/s12933-019-0928-8)

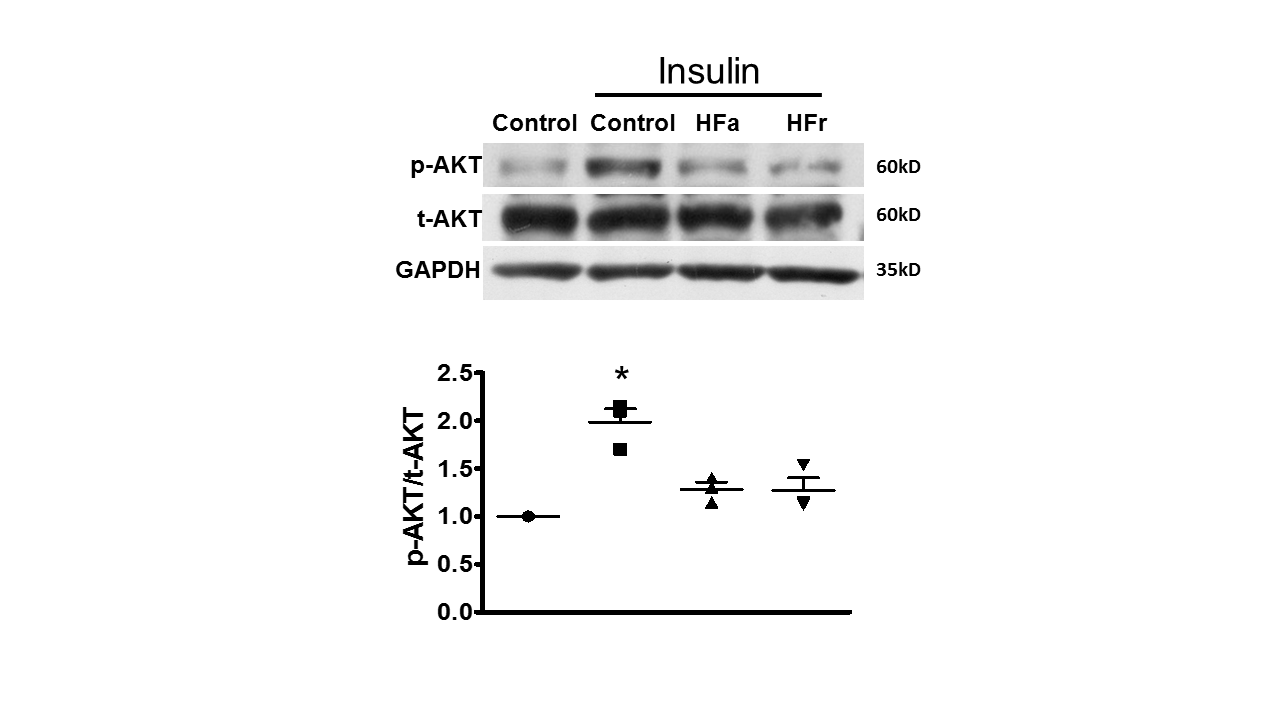

Supplement: Supplementary file 1 — Additional file 1: Figure S1. Expression of phosphorylated and total AKT in insulin-treated high-fat (HFa) and high-fructose/cholesterol (HFr) diet-fed rat atria. Representative examples (upper panel) and mean ± SE of the relative expression levels of each protein quantified by densitometry and normalized to the control level, which was set at 1.0 (lower panel). Each value represents the mean ± SE of three independent experiments. [file 12933_2019_928_MOESM1_ESM.tif]

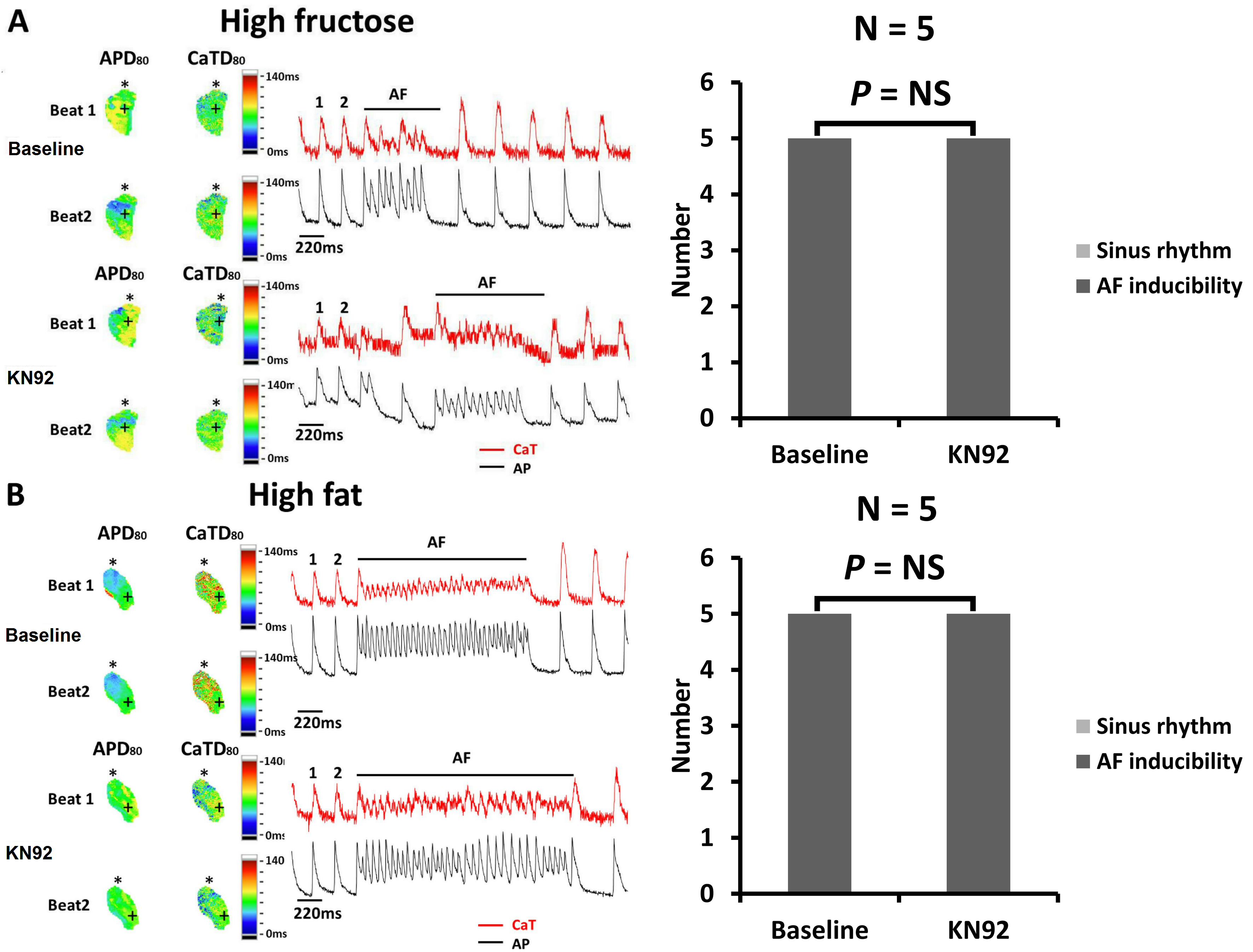

Supplement: Supplementary file 3 — Additional file 3: Figure S2. Effects of KN-92 in atrial fibrillation (AF) induced by short pacing cycle length (PCL) in high-fat (HFa) and high-fructose/cholesterol (HFr) diet-fed rats. The figure shows representative optical membrane voltage (AP) and calcium transient (CaT), action potential duration at 80% repolarization (APD80), and CaT duration at 80% repolarization (CaTD80) maps of right atrial appendages (RAAs) in (A) one HFr diet-fed rat and (B) one HFa diet-fed rat at short PCLs before and after application of KN-92 (1 µmol/L), respectively. None of the HFr or HFa rat atria (n = 5 for each group) exhibited decrease or increase in AF duration after KN-92 infusion. [file 12933_2019_928_MOESM3_ESM.tif]
