# Supplementary material for: Atrial fibrillation and its arrhythmogenesis associated with insulin resistance
Source: Cardiovasc Diabetol. 2019 Sep 26;18:125. doi: 10.1186/s12933-019-0928-8 (PMC6761716; doi:10.1186/s12933-019-0928-8)
Supplement: Supplementary file 2 — Additional file 2: Table S1. Cellular electrophysiology in HFa and HFr rats. [file 12933_2019_928_MOESM2_ESM.doc]

**Additional file 2: Table S1. Cellular electrophysiology in HFa and HFr rats**

|  | **RMP (mV)** | **APA (mV)** | **APD_20_ (ms)** | **APD_50_ (ms)** | **APD_90_ (ms)** |
| --- | --- | --- | --- | --- | --- |
| **Control** | 81 ± 2 | 101.8 ± 3.2 | 5.8 ± 1.0 | 14.8 ± 3.5 | 53.5 ± 8.3 |
| **HFa** | 80 ± 4 | 104.4 ± 3.9 | 5.8 ± 1.3 | 13.3 ± 2.6 | 50.3 ± 6.9 |
| **HFr** | 77 ± 2 | 97.4 ± 5.3 | 5.0 ± 0.6 | 11.5 ± 1.0 | 51.0 ± 8.7 |

Data are presented as mean ± SE.

Abbreviations: **HFa** = high-fat diet; **HFr** = high-fructose/cholesterol diet; **RMP** = resting membrane potential; **APA** = action potential amplitude; **APD20, APD50, APD90** = action potential duration at 20%, 50%, and 90% repolarization, respectively.

n = 4 atrial myocytes for each group.
